# Randomized controlled trial of artesunate or artemether in Vietnamese adults with severe falciparum malaria

**DOI:** 10.1186/1475-2875-9-97

**Published:** 2010-04-15

**Authors:** Nguyen H Phu, Phung Q Tuan, Nicholas Day, Nguyen TH Mai, Tran TH Chau, Ly V Chuong, Dinh X Sinh, Nicholas J White, Jeremy Farrar, Tran T Hien

**Affiliations:** 1 Hospital for Tropical Diseases, 190 Ham Tu, Quan 5, Ho Chi Minh City, Vietnam; 2 Oxford University Clinical Research Unit, Hospital for Tropical Diseases, Wellcome Trust Major Overseas Programme 190 Ham Tu, Quan 5, Ho Chi Minh City, Vietnam; 3 Nuffield Department of Clinical Medicine, John Radcliffe Hospital, Oxford, OX3 9DU, UK

## Abstract

**Background:**

Both artemether and artesunate have been shown to be superior to quinine for the treatment of severe falciparum malaria in Southeast Asian adults, although the magnitude of the superiority has been greater for artesunate than artemether. These two artemisinin derivatives had not been compared in a randomized trial.

**Methods:**

A randomized double blind trial in 370 adults with severe falciparum malaria; 186 received intramuscular artesunate (2.4 mg/kg immediately followed by 1.2 mg/kg at 12 hours then 24 hours then daily) and 184 received intramuscular artemether (3.6 mg per kilogram immediately followed by 1.8 mg per kilogram daily) was conducted in Viet Nam. Both drugs were given for a minimum of 72 hours.

**Results:**

There were 13 deaths in the artesunate group (7 percent) and 24 in the artemether group (13 percent); P = 0.052; relative risk of death in the patients given artesunate, 0.54; (95 percent confidence interval 0.28-1.02). Parasitaemia declined more rapidly in the artesunate group. Both drugs were very well tolerated.

**Conclusions:**

Intramuscular artesunate may be superior to intramuscular artemether for the treatment of severe malaria in adults.

## Background

Over the past 25 years, the artemisinin derivatives have been used for the treatment of severe malaria. They are the most rapidly acting and potent of all the anti-malarial drugs. They can be given once daily and are safer and easier to administer compared to quinine. Artemisinin derivatives are now part of recommended first-line combination treatment for uncomplicated falciparum malaria everywhere in the tropical world [1]. Recently, the largest ever randomized trial in severe malaria compared intravenous artesunate with intravenous quinine. It enrolled 1,461 patients with severe falciparum malaria in Southeast Asia before it was stopped early by the data and safety monitoring committee. Artesunate reduced mortality by 35% compared to quinine. The number needed to treat to save one life ranged from 11 to 20 [2]. As a result the new World Health Organization recommended parenteral artesunate as the first choice anti-malarial treatment of severe malaria in low transmission areas [1]. But the recommendation for higher transmission areas (i.e. much of Africa) has remained either artemether, artesunate or quinine. The reason for this continued equipoise is that there maybe important differences in the clinical manifestations and evolution of severe malaria and the treatment responses in African children compared with adults in Southeast Asia. Notably, in a meta-analysis of randomized trials in severe malaria, intramuscular injection of the oil-based artemisinin derivative, artemether, did reduce mortality in Southeast Asian adults compared with quinine, but it did not do so in African children [3]. This raised the possibility that artemether was not inferior to artesunate for the treatment of severe falciparum malaria in Southeast Asian adults. The largest study from South East Asia in this series was a randomized comparison of artemether and quinine, which enrolled 560 adults and was conducted in the Hospital for Tropical Diseases, Ho Chi Minh City, Vietnam [4]. This study reports a subsequent study from this centre in which artemether was compared with artesunate.

## Methods

This was a single centre double blind comparison of intramuscular artesunate and intramuscular artemether in patients admitted to the Hospital for Tropical Diseases, Ho Chi Minh City with severe falciparum malaria. It was conducted between May 1996 and June 2003. This study was approved by the Ethical and Scientific Committee of the Hospital for Tropical Diseases, Ho Chi Minh City. All study procedures were essentially as reported in previous studies performed in the Hospital for Tropical Diseases in patients with severe malaria [4].

### Entry criteria

Patients were included in the study if their peripheral-blood smears had asexual forms of *Plasmodium falciparum *and had at least one of the following severe complications: cerebral malaria (Glasgow Coma Score was less than 11), renal acute failure (oliguria and serum creatinine > 250 μmol/L), jaundice (total serum bilirubin > 50 μmol/L) with a parasite count of more than 100,000/μL or with serum creatinine > 250 μmol/L, hypoglycaemia (blood glucose < 2.2 mmol/L), anaemia (haematocrit < 20%) with a parasite count of more than 100,000/μL, hyperparasitaemia (parasite count > 500,000/μL), hyperlactataemia (plasma lactate > 4 mmol/L), metabolic acidosis (standard base excess > - 5 mmol/L, base deficit < 10 mmol/L) and shock (systolic blood pressure < 80 mmHg with cool extremities).

Informed consent was provided by all studied patients (or accompanying relative if patient was unconscious).

Patients were not included if they were younger than 14 years, were pregnant in the first trimester, were known intravenous drug abusers, had received more than 3 g of quinine or 2 doses of any artemisinin derivatives in the previous 48 hours before admission, had a past history of allergy to any artemisinin derivatives, or if known to be HIV positive.

### Management, clinical and parasitological monitoring

A full history was recorded from either the patient or their relatives. Patients were examined fully on admission. Baseline blood samples were taken for full blood count, biochemistry tests (blood urea nitrogen, serum creatinine, serum bilirubin, aspartate aminotransferase, alanine transaminase, plasma lactate), blood culture and malaria parasite counts. Arterial pH and blood gases were also measured. Chest X-ray was also performed on admission. Hydration and neurological status were fully assessed. A urinary catheter was inserted if necessary. Lumbar puncture was done if Glasgow Coma Score was below 14. A Gram stain and bacterial culture was performed on all cerebrospinal fluid samples and was analyzed for protein, glucose, lactate levels and cell counts.

Patients were treated as per standard recommendations and as previously reported [4]. Briefly blood transfusion was administered if the haematocrit was below 20 percent, a bolus of 50 ml of 30 percent dextrose was administered if hypoglycaemia was identified and a single dose of phenobarbital (3.5 mg/kg) was give for all patients with a GCS ≤ 11. Haemofiltration was performed in patients who developed acute renal failure or severe lactic acidosis and patients ventilated if they developed respiratory failure or a syndrome consistent with adult respiratory distress syndrome (ARDS). Haematocrit and peripheral blood smears were performed every four hours for the first 24 hours and every six hours until asexual stages of parasite were not detected in three consecutive smears. Clinical observations were recorded on standard case record forms every 4 hours for the first 24 hours, and every 6 hours subsequently until discharged.

### Anti-malarial treatment

Patients were randomized to treatment with either intramuscular artesunate or intramuscular artemether. Artesunate (Guilin No 2 Pharmaceutical Factory, Guangxi, People's Republic of China) was given in a dose of 2.4 mg/kg body weight on admission, then 1.2 mg/kg was given daily. Artemether (50 mg per millilitre; Kunming Pharmaceutical Company, Kunming, People's Republic of China) was given in a dose of 3.2 mg/kg body weight on admission, followed by 1.6 mg/kg daily. Both drugs were given intramuscularly to the anterior thigh until oral medication could be taken reliably. Oral artesunate was given in a dose of 2 mg/kg/day to complete a total course of seven days (including parenteral doses), providing a total cumulative dose between 17 and 18 mg/kg over seven days.

Treatment failure was considered if parasitaemia did not fall by >75% of the admission value within 48 hours and intravenous quinine (20 mg/kg followed by 10 mg/kg 8 hourly) was given.

### Randomization, concealment and treatment allocation

The randomization was generated from random number tables. Labels with the name of drug for each patient were put in coded sealed opaque envelopes, and the envelopes were randomized in blocks of 20. Once a patient was enrolled in the study the envelope was opened. An independent team of nurses, not otherwise involved in the study or responsible for the care of these patients, open the envelope, randomized the patient and prepared the injection. Neither the treating physicians, study doctors and nurses, or patients knew which anti-malarial drugs was administered.

### Statistical analysis

The study was reviewed continuously by an external monitor. Results were entered into a database (Microsoft Excel, Microsoft Corp, USA). Categorical data were analyzed with a statistical software package (Stata 10, StataCorp, Texas, USA). Chi- square or Fisher's exact test was used to compare proportions as appropriate. Risk verification was expressed with odds ratio (OR) and 95% confidence interval (CI). The original sample size was calculated on an assumption of a 20% mortality in the artemether arm and a 50% reduction with artesunate. Assuming 80% power and 0.05 significance the study would have needed to recruit 400 patients into each arm (allowing for 10% loss to follow up). From the year 2000 onwards, the number of patients with severe malaria declined dramatically and a decision was made prior to unblinding to stop the study on the grounds of future futility. At this stage 370 patients had been recruited, 186 in the artesunate arm and 184 in the artemether arm. Assuming a 20% of mortality in the artemether arm the study at the time of stopping therefore had a power of 70% to demonstrate an approximate 50% reduction in absolute mortality.

## Results

Between May 1996 and June 2003, 370 patients were recruited in the study. There was a gradual rise in number of severe malaria patients admitted to the HTD with peak in 2000, but after this date there was a sharp drop of severe malaria patients and a dramatic decline in the number of malaria patients admitted in the subsequent two years. Because of this sustained fall in the number of patients, a decision was made by the investigators on the grounds of future futility to terminate the study. This was done prior to unblinding of the treatment allocation. The baseline characteristics of patients on admission are shown in Table 1. Both admissive clinical parameter and laboratory investigation are similar among two randomized treatment groups.

**Table 1:** Baseline characteristics of patients, according to treatment group.

Parameters	Number (ARTS/ARTM)	Artesunate (ARTS) n = 186	Artemether (ARTM) n = 184	P value
Age (years) Median (Range)		32(15-74)	32.5(15-77)	0.33

Sex - Male/Female		133/53	142/42	0.21

Previous treated with quinine for current malaria contracted: no. of patients (%)		11(5.9)	15(8.2)	0.4

Previously treated with artemisinin derivatives for current malaria episode: no. of patients (%)		114(61.3)	105(57.1)	0.41

Convulsions: no. of patients (%)		13(7.0)	14(7.6)	0.82

Temperature (°C) Median (Range)		38.1(36.5-40.5)	38.0(36.0-41.0)	0.75

Pulse (rate/min) Median (Range)		100 (68-150)	100 (60-151)	0.30

Respiratory rate (/min) Median (Range)		28 (12-60)	28 (18-68)	0.27

Glasgow Coma Score Median (Range)		11 (3-15)	11 (3-15)	0.61

Glasgow Coma score less than 11: no. of patients (%)		85(45.7)	87(47.3)	0.76

Shock: no. of patients (%)		6(3.2)	10(5.4)	0.30

Hypoglycaemia: no. of patients (%)		1(0.5)	0(0)	1.0

Haematocrit (%) Median (Range)		32 (10-50)	31 (8-53)	0.42

Parasite count (×10^3^/mm^3^) Median (Range)		80.7(0.02-3471)	70.5 (0.02-3534)	0.75

White cell count (×10^3^/mm^3^) Median (Range)	173/171	7.93 (1.1-47.6)	8.40 (1.7-27.0)	0.45

Serum creatinine (mg/dL) Median (Range)	179/176	1.8 (0.6-9.5)	1.7 (0.6-17.0)	0.68

Total bilirubin (mg/dL) Median (Range)	124/120	5.7 (0.6-39.0)	5.0 (0.6-38.8)	0.40

ALT (U/L) Median (Range)	155/161	70 (10-1900)	71 (6-414)	0.45

Lactate (mmol/L) Median (Range)	168/166	3.2 (0.5-17.7)	3.6 (0.5-16.8)	0.85

### Outcome

The overall mortality rate was ten percent (37 of 370 patients). There were 13/186 (7%) deaths in the artesunate (ARTS) treated patients compared with 24/184 (13%) deaths in the artemether (ARTM) treated patients unadjusted relative risk of death for artesunate = 0.54, 95% CI: 0.28 - 1.02, p = 0.052 (see Table 2). For the cerebral malaria subgroup of patients, there was no significant difference in this outcome: the unadjusted mortality in the ARTS group was 12.9 percent (11/85) and was 13.8 percent (12/87) in the ARTM group (RR for artesunate = 0.94, 95% CI: 0.44 - 2.01, p = 0.87). Further exploration in factors associated with fatal outcome using multiple logistic regression (see Table 3), patients who received intramuscular artesunate had an adjusted odds ratio of 0.41 (95% CI: 0.16 - 1.06, p = 0.06) risk of death compared to treatment with artemether. Other important contributors associated with death were admission to hospital after year 2000, increased plasma lactate on admission, decreased Glasgow coma score, previous treatment with artemisinin derivatives and rise of white cell count in the peripheral blood. No significant difference was found in of the rate of complications between the groups except for the development of coma after receiving treatment. The ARTS group was associated with a lower risk (7/94, 6.9%) of developing coma than the ARTM group (15/82, 15.5) RR = 0.45 CI 95%: 0.19 - 1.05, p = 0.056. This may help explain why the mortality in the former subgroup was also lower than the latter (2/99 vs. 12/85, RR = 0.16 CI 95% 0.04 - 0.70, p = 0.004).

**Table 2:** Outcomes after treatment with intramuscular artesunate or artemether.

Outcomes	ARTS n = 186	ARTM n = 184	Relative Risk (95% CI)	P value
Mortality (%)	13(7.0)	24(13.0)	0.54(0.28-1.02)	0.052

Convulsions (n)	7(3.8)	9(4.9)	0.77(0.29-2.02)	0.59

Required blood transfusion (%)	69(37.1)	69(37.5)	0.99(0.76-1.29)	0.94

Renal impairment (%)	111(59.7)	105(57.1)	1.05(0.88-1.24)	0.61

Renal failure (%)	85(45.7)	85(46.2)	0.99(0.79-1.23)	0.92

Required dialysis (%)	52(28)	54(29.3)	0.95(0.69-1.31)	0.77

Hypoglycaemia (%)	7(3.8)	9(4.9)	0.77(0.29-2.02)	0.59

Spontaneous bleeding (%)	26(14)	35(19)	0.73(0.46-1.17)	0.19

Shock (%)	22(11.8)	26(14.1)	0.84(0.49-1.42)	0.51

Concomitant infection (%)	66(35.5)	75(40.8)	0.87(0.67-1.13)	0.30

Jaundice (%)	144(77.4)	144(78.3)	0.99(0.89-1.1)	0.85

Pulmonary oedema (%)	10(5.4)	13(7.1)	0.76(0.34-1.69)	0.5

**Table 3:** Multivariate logistic regression analysis of factors associated with death in severe falciparum malaria patients.

Factors	Adjusted Odds Ratio (95% CI)	P* value	Notes
IM artesunate treatment	0.41(0.16 - 1.06)	0.066	60 out of 370 were not included (30 in ATS and 30 in ATM) due to missing of lactate and/or WBC count)
	
Year of study (after 2000)	0.14(0.03 - 0.71)	0.017	
	
Each unit higher in Glasgow Coma score	0.82(0.72 - 0.93)	0.002	
	
Unit increase in admission plasma lactate	1.28(1.15 - 1.43)	< 0.001	
	
Each 10^3 ^WBC/mm^3 ^rise	1.1(1.02 - 1.18)	0.017	
	
Pre-treatment with artemisinin derivatives	0.29(0.11 - 0.82)	0.019	

Forward stepwise on conditioned of likelihood change logistic regression (p to enter = 0.075, p to remove = 0.15)

### Causes of death

The causes of death in severe malaria are often not confined to any single factor or complication, as multiple organ disorder often developed before death. Of the 37 fatal cases, 25 had acute renal failure (24 of this group underwent dialysis); 28 cases had persistent intractable shock; 20 cases had spontaneous bleeding mostly from gastro-intestinal tract; 12 cases had suspected nosocomial co-infection of lung and urinary tract; and eight cases had pulmonary oedema.

### Recovery

**Figure 1:**
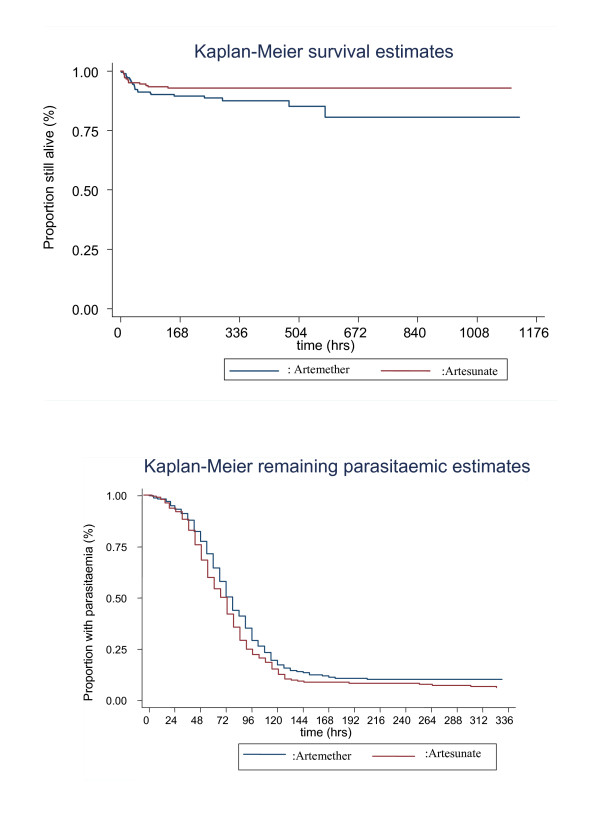
**Kaplan-Meier plots of overall survival and remaining parasitaemic estimates**.

Assessment of standard markers of recovery from severe malaria did not reveal any significant differences between the treatment groups (see Table 4). However, the ARTS group was associated with a quicker initial clearance of parasite from the peripheral blood (median time to 90% parasite clearance (IQR) (hrs): 16 (12-30) in ARTS and 24 (12-36) in ARTM) (see Table 4 and Figure 1).

**Table 4:** Assessment of recovery after treatment with intramuscular artesunate or artemether.

Parameters	ARTS n = 186	ARTM n = 184	P value*
		
	hours		
Time to parasite clearance			
Decrease to 50% of admission value			0.007
Median (Range)	8 (4-42)	8 (2-60)	
Decrease to 90% of admission value			0.004
Median (Range)	16 (4-102)	24 (2-152)	
Total clearance			0.97
Median (Range)	72 (7-330)	72 (2-204)	

Resolution of fever			
Time first temperature < 37.5°C			0.99
Median (Range)	30 (0-768)	20 (0-648)	
Time of fever clearance			0.27
Median (Range)	108 (0-888)	108 (0-1088)	

Time to recovery from coma			
Time to Glasgow Coma score of 8^†^			0.55
Median (Range)	48 (4-2136)	54 (2-2232)	
Time to Glasgow Coma score of 11^‡^			0.44
Median (Range)	42 (4-2136)	42 (2-2232)	
Time to Glasgow Coma score of 15^¶^			0.11
Median (Range)	60 (4-2136)	72 (2-2232)	

Time to physical recovery			
Time before patient able to drink			
Median (Range)	30 (0-2136)	42 (0-2232)	0.2
Time before patient able to eat			
Median (Range)	42 (0-2136)	46 (0-2232)	0.18
Time before patient able to sit			
Median (Range)	78 (0-2136)	90 (0-2232)	0.32
Time before patient able to stand			
Median (Range)	96 (0-2136)	114 (0-2232)	0.66
Time before patient able to walk			
Median (Range)	102(0-2136)	120 (0-2232)	0.64
Time before patient able to leave hospital			
Median (Range)	264(7-2136)	288 (2-2232)	0.68

* Log-rank test

† Only patients who decreased in Glasgow Coma score below 8 were included (130 pts, 58 in ATS group and 72 in ATM group)

‡ Only patients who decreased in Glasgow Coma score below 11 were included (204 pts, 93 in ATS group and 111 in ATM group)

¶Only patients who decreased in Glasgow Coma score below 15 were included (274 pts, 134 in ATS group and 140 in ATM group)

### Haematologic recovery

There was no significant difference between two groups in fall of haematocrit after baseline (median percentage reduction of admissive haematocrit in the ARTS group: 23.1%, IQR (14.3-35.1), range (0-70%) and in the ARTM group: 21.4%, IQR (12.9-34.7), range (0-70%) p = 0.25), or in the number of times required blood transfusions (p = 0.19).

### Adverse effects

After treatment hypoglycaemia was documented in three patients in the artesunate group and 5 in the artemether group. No local abscesses, urticaria or rashes were found in both groups. There were no severe unexpected adverse events.

## Discussion

The treatment of severe malaria has changed in recent years with the introduction of the artemisinin derivatives. Until recently, artemether was favoured by World Health Organization and, therefore, most studies had been conducted with this derivative. In an individual patient data meta-analysis of randomized trials in severe falciparum malaria artemether was superior to quinine in Southeast Asian adults, but not in African children [3]. Unfortunately intramuscular artemether was probably not the best artemisinin formulation to choose; it is an oil-based preparation that releases the drug slowly and erratically particularly in patients with severe acidosis [5,6]. In contrast to intramuscular artemether, intramuscular artesunate is almost instantly bioavailable, and absorbed reliably and rapidly with peak concentrations occurring within thirty minutes [6-8]. Moreover, artesunate is a more potent anti-malarial; artesunate and its main active metabolite dihydroartimisinin have relative in vitro potencies, in comparison to artemether, of 2.9 and 4.0 respectively [9]. A recent large multicentre trial from Southeast Asia demonstrated that artesunate reduced mortality by 35% compared with quinine [2]. This left the role of artemether uncertain - was it as good as artesunate despite the pharmacokinetic drawbacks, or was it inferior? This trial set out to answer this question.

The ARTS group was associated with lower risk (7/101, 6.9%) of developing coma after initiation of therapy than the ARTM group (15/97, 15.5) RR = 0.45 CI 95%: 0.19 - 1.05, p = 0.056. The parasite clearance times were faster in the ARTS treated patients (median 90% reduction PCT (IQR) (hrs): 16 (12-30) in ARTS and 24 (12-36) in ARTM). These data may be explained by the pharmacokinetic properties of artesunate compared with artemether and the faster bioavailaibilty of the active metabolites with artesunate especially in severely ill patients with marked metabolic acidosis. It also suggests that the first few hours of effective treatment are very important and the faster the active drug can be got to the site of action (the blood) the better. As outlined above after intramuscular injection concentrations of artesunate peaked within 30 minutes of injection, and artesunate was hydrolysed immediately and completely to the biologically active dihydroartemisinin (DHA). Maximum artemether concentrations occurred at a median of 10 hours and was associated with a much more erratic and slower conversion to DHA. Importantly, in a particular subgroup of patients with severe metabolic acidosis the absorption and conversion to DHA was erratic, limited and delayed. This hypothesis would be consistent with previous pharmacokinetic work undertaken at the Hospital for Tropical Diseases [4]. Getting active drug effective against all stages of the parasites (and thereby reducing or preventing maturation and reducing sequestration) into the patient quickly seems crucial. When faced with a patient with severe malaria the first few minutes and hours may make a significant difference to their subsequent chances of survival. Perhaps it is time to learn the lessons from the cardiologists and aim to administer the most effective, fast-acting anti-malarial drug into patients as an emergency in the same way as thrombolytic therapy is administered urgently in patients with myocardial infarction, where delays of minutes can make a huge difference to mortality and preservation of myocardium. Getting patients treated early and then referred quickly through the health care system as appropriate is crucial. Once in a hospital, the ability to triage and administer fast-acting, potent parenteral drugs would seem essential to prevent the further maturation of parasites, reduce sequestration and hence prevent consequent complications.

The trial was overtaken by events. Improved malaria control in Vietnam led to a dramatic decline in the incidence of severe malaria. The Hospital for Tropical Diseases is a referral hospital, which in the early 1990s was receiving approximately three to five patients each day with severe malaria. A decade later this had fallen to approximately one patient per month. This was insufficient to sustain this trial and so the trial was terminated before reaching the pre-defined end points. The trial was stopped with the investigators blind to the allocations and prior to any analysis of the results.

The result after 370 patients had been randomized showed a borderline difference in favour of artesunate (unadjusted relative risk of death for artesunate = 0.54, 95% CI: 0.28 - 1.02, p = 0.052). The superiority of artesunate over artemether is supported by the larger differences in mortality rates when artesunate was compared with quinine (35% reduction in mortality), versus artemether (a 26% reduction), although these trials were conducted in different sites, with different protocols and different drug dosages, so are not strictly comparable [2,3]. There is probably little to choose intrinsically between these two derivatives in terms of anti-malarial pharmacodynamics, and so the superiority of artesunate is likely to derive from its more rapid, and more reliable absorption and conversion to the active compound dihydroartemisin following intramuscular injection particularly in those severely ill patients with marked acidosis.

Both drugs were very well tolerated with little or no discomfort at the injection site, no serious local or systemic reactions, a low rate of hypoglycaemia (presumably disease related), and no neurological sequelae. Although artemether is easier to administer than artesunate, which is an advantage in busy epidemic settings, it is probably an inferior drug. Artesunate, administered as an emergency, should be the treatment of choice for severe falciparum malaria.

The patients recruited to this trial all had severe falciparum malaria as defined by the World Health Organization. In terms of standard prognostic clinical markers of severity these patients were more severe than previously reported patients recruited into similar studies in Vietnam or compared with those patients entered into regional trials (data not shown) [2,4]. In this trial, however, the overall mortality was 10% (7% in the ARTS group and 13% in the ARTM group), which was lower than anticipated at the start of the trial when a mortality of 20% in the ARTM was expected from experience and reported studies. The reduction in mortality in patients with severe falciparum malaria in this setting is inevitably multifactorial and it is only possible to hypothesize on why such a reduction has occurred. The general intensive care of patients at HTD and across Vietnam has improved over the last two decades with improvements in nursing and medical care, earlier identification of renal failure, acidosis and respiratory failure and increasingly availability and use of haemofiltration and mechanical ventilation. These improvements and the increasing use of the parenteral artemisinin derivatives have lead to a reduction in the mortality in severe malaria patients at HTD from 40% (over 70% mortality in those with renal failure) in 1991 to approximately 5% in 2009 (15% mortality rate in those with patients with renal failure). This is despite an apparent increase in the severity of the patients admitted in recent years. The combination of urgent treatment with potent anti-malarial drugs and improved intensive care has had a profound impact on the mortality rates in severe malaria in this setting.

## Conclusion

Intramuscular artesunate may be superior to intramuscular artemether for the treatment of severe malaria in adults. Artesunate, administered as an emergency, should be the treatment of choice for severe falciparum malaria.

## Abbreviations

ARTS: artesunate; ARTM: artemether; DHA: dihydroartemisinin.

## Competing interests

The authors declare that they have no competing interests.

## Authors' contributions

NHP, ND, NW, JF and TTH participated in the design of the study. NHP, NTHM, TTHC, LVC, DXS, TTH and JF were involved in recruiting the patients and collecting clinical data. PQT performed the statistical analysis. NHP, ND, NW, JF and TTH were primarily responsible for drafting the manuscript. All authors approved the final version.
